# The performance of serious games for enhancing attention in cognitively impaired older adults

**DOI:** 10.1038/s41746-023-00863-2

**Published:** 2023-07-08

**Authors:** Alaa Abd-alrazaq, Israa Abuelezz, Eiman Al-Jafar, Kerstin Denecke, Mowafa Househ, Sarah Aziz, Arfan Ahmed, Ali Aljaafreh, Rawan AlSaad, Javaid Sheikh

**Affiliations:** 1 AI Center for Precision Health, Weill Cornell Medicine-Qatar, Doha, Qatar; 2 Division of Information and Computing Technology, College of Science and Engineering, Hamad Bin Khalifa University, Qatar Foundation, Doha, Qatar; 3 Kuwait Health Informatics Association, Kuwait, Kuwait; 4 Institute for Medical Informatics, Bern University of Applied Science, Bern, Switzerland; 5 Department of Management Information Systems, School of Business, Mutah University, Karak, Jordan

**Keywords:** Dementia, Geriatrics

## Abstract

Attention, which is the process of noticing the surrounding environment and processing information, is one of the cognitive functions that deteriorate gradually as people grow older. Games that are used for other than entertainment, such as improving attention, are often referred to as serious games. This study examined the effectiveness of serious games on attention among elderly individuals suffering from cognitive impairment. A systematic review and meta-analyses of randomized controlled trials were carried out. A total of 10 trials ultimately met all eligibility criteria of the 559 records retrieved. The synthesis of very low-quality evidence from three trials, as analyzed in a meta-study, indicated that serious games outperform no/passive interventions in enhancing attention in cognitively impaired older adults (*P* < 0.001). Additionally, findings from two other studies demonstrated that serious games are more effective than traditional cognitive training in boosting attention among cognitively impaired older adults. One study also concluded that serious games are better than traditional exercises in enhancing attention. Serious games can enhance attention in cognitively impaired older adults. However, given the low quality of the evidence, the limited number of participants in most studies, the absence of some comparative studies, and the dearth of studies included in the meta-analyses, the results remain inconclusive. Thus, until the aforementioned limitations are rectified in future research, serious games should serve as a supplement, rather than a replacement, to current interventions.

## Introduction

The pace of global population aging is continuously increasing. According to the United Nations’ World Population Prospects 2022 report, globally, there will be 771 million elder people (65 years or over) by the end of 2022; this is expected to increase to 994 million by 2030, a 12 percent increase^1^. An improvement in life expectancy generally correlates with better public health services, sanitation, and quality of life. However, a growing aging population is associated with a decline in physical activities, psychological status, and cognitive capacity^2^.

Cognitive impairment is one of the main health issues that affect older adults. Cognitive impairment is described as the deterioration of cognitive abilities such as memory, processing speed, executive functions, and intelligence. Examples of cognitive disorders include dementia, Alzheimer’s disease (AD), attention deficit disorder, and mild cognitive impairment (MCI)^2,3^. As estimated by the Alzheimer’s Association, MCI affects about 15 to 20 percent of the US elder population. Globally, about 55 million have dementia in 2020, and it is estimated to reach 78 million in 2030^4^.

Attention is a cognitive function described as the process of noticing ones surrounding environment and processing this information to execute functions of daily living such as planning, organization, and management^5,6^. Based on Sohlberg and Mateer’s model^7^, attention can be divided into five types: focused attention (this refers to the capacity to respond specifically to distinct visual, auditory, or tactile stimuli), sustained attention (this involves the ability to maintain alertness over a period of time), alternating attention (this requires the capability to discontinue one task to engage in another, and then revert back to the initial task), selective attention (this represents goal-oriented concentration on task-related information while disregarding other irrelevant details), divided attention (this encompasses the ability to concurrently carry out two or more tasks or process multiple sources of information). As people grow older, attention function deteriorates gradually.

While the decline in attention associated with aging cannot be reversed, it can be effectively managed with a range of therapeutic strategies and adaptations. These approaches can significantly enhance the quality of life for older individuals dealing with attention issues^8,9^. Fortunately, the older brain retains plasticity abilities, which may help reduce the negative effects of aging on attention through cognitive training and exercise^9^. Non-pharmacological interventions have been used for promoting attention and other cognitive functions among the elderly population^10^. Non-pharmacological interventions causing cognitive stimulation include cognitive training programs, cognitive behavioral therapy, nutrition, social therapy, physical exercise, psychological therapy, and serious games^11^.

Serious games are defined as interactive games designed for education, simulation, or training purposes, as opposed to games that are purely used for entertainment purposes. They have proven effective in enhancing various cognitive abilities among older individuals^12–17^. Exergames and cognitive training games are prevalent categories within the realm of serious games. Exergames integrate physical exercises into their intended gameplay, offering a unique interactive experience^14^. On the other hand, cognitive training games are a type of video games designed to engage and enhance cognitive abilities like executive function and attention^14^. It has been estimated that the global market for serious games was $5.94 billion in 2020. Projections indicate a significant surge by 2030, with expectations reaching approximately $32.72 billion^18^. Serious games for seniors are developed with special features. In particular, there must be an emphasis on simple operation, customization capabilities, intuitive and easy-to-remember game mechanics, and game principles.

There has been an increased interest in serious games for improving the health and well-being of elderly people over the last few years^19–23^. However, there is a lot of speculation in regard to the potential of serious games in enhancing attention in cognitively impaired older individuals with fragmented evidence of their effectiveness.

The role of serious games in enhancing attention has been examined in numerous studies. However, despite several systematic reviews that have synthesized the findings of these studies, they possess certain limitations: (1) the reviews concentrated largely on healthy elderly individuals rather than those dealing with cognitive impairment^11,24–27^, (2) these reviews incorporated preliminary randomized controlled trials (RCTs)^11,28^, (3) previous reviews did not assess the quality of evidence^11,24,27,28^, (4) these reviews solely concentrated on a particular kind of serious games, such as cognitive training games^24,25^ or exergames^11,27^, and (5) these reviews did not compare the impact of serious games with a specific control group (for example, no intervention, traditional exercises, conventional cognitive training)^11,24,27,28^. To achieve this, our review included only RCTs that targeted elderly individuals with cognitive disorders. Furthermore, this review considered the quality of the evidence. Moreover, this study encompassed all varieties of serious games and compared their influence with a specific control group.

## Results

### Search results

**Fig. 1:**
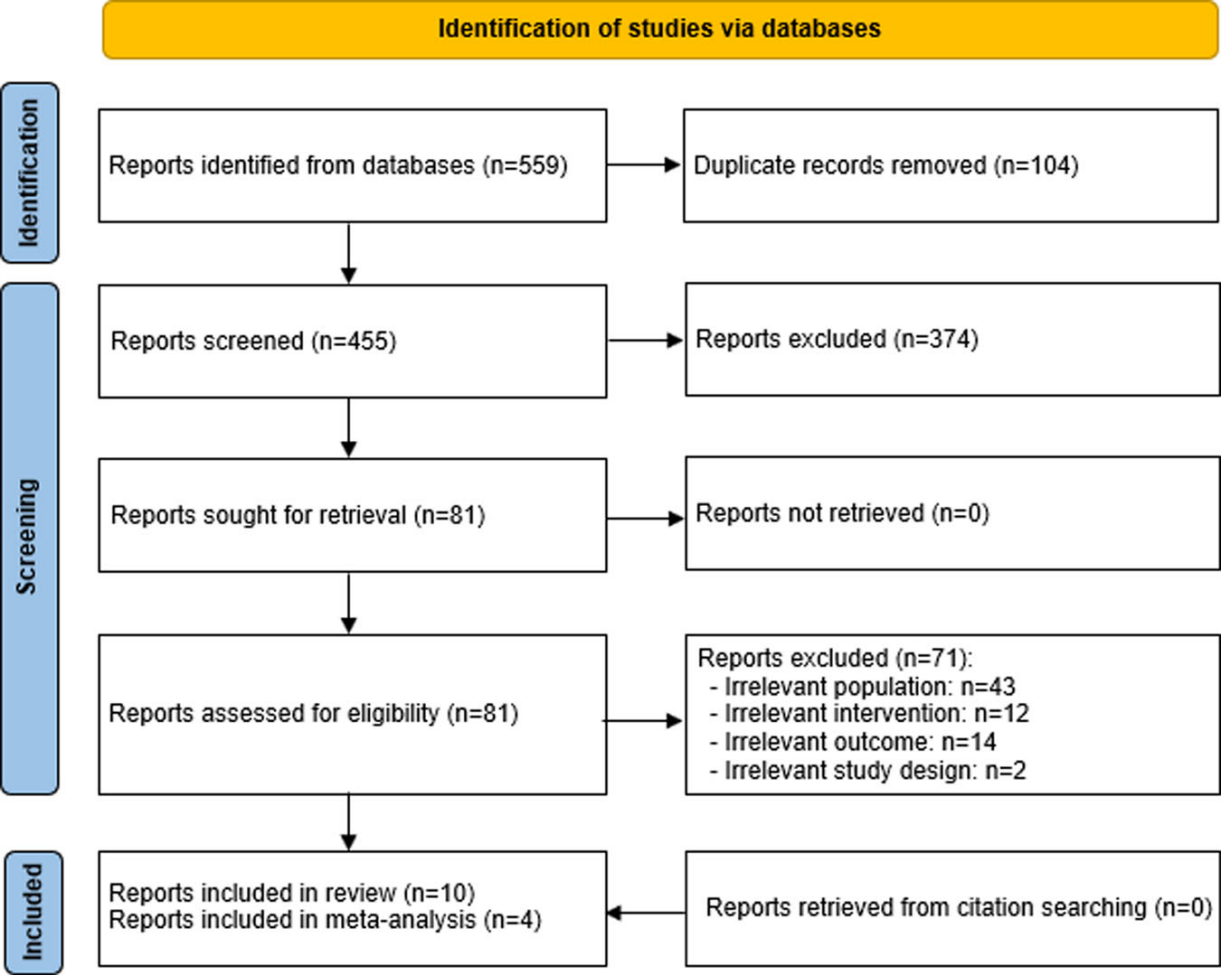
Flow diagram of the study selection process. A total of 559 publications were retrieved by searching all databases. Of these, 104 duplicates were removed. Screening titles and abstracts of the remaining publications led to excluding 374 citations. By reading the full text of the remaining 81 publications, we excluded 71 publications. No additional studies were found by backward and forward list checking. The current study included a total of 10 RCTs. However, four of these papers were included in the meta-analyses.

After searching the predefined databases, 559 records were retrieved, as shown in Fig. 1. One hundred four duplicates were removed from these records using the EndNote X9. After examining the titles and abstracts of the remaining records, 374 citations were removed. A total of 71 studies were removed after scanning the full texts of the remaining 81 publications for various reasons shown in Fig. 1. No additional studies were found by backward and forward list checking. The current study included a total of 10 RCTs^29–38^. However, four of these papers were included in the meta-analyses^29–32^.

### Characteristics of included studies

The identified studies were conducted in eight different countries and published between 2012 and 2019 (Table 1). Except for one book chapter, all the studies included were journal articles. In all studies, parallel RCTs were used. The samples of the studies ranged in size from 20 to 209, with an average of 74.1. The average mean age of participants in the included studies is 72.2 years, with ages spanning from 66 to 83.1 years. The representation of males in the included trials varied between 21.5 and 70%, averaging 45.3%. The MMSE score, recorded in eight studies, fluctuated between 10.2 and 27.3, with a mean score of 22.6. The most prevalent condition among the study participants was MCI, identified in eight studies. In terms of recruitment, eight studies recruited participants from clinical environments, whereas the other two studies recruited participants from the general community.

**Table 1:** Characteristics of studies and population.

Study [Ref]	Year	Country	Publication type	RCT type	Sample size	Mean age	Male (%)	MMSE score	Health condition	Setting
Cavallo^1^	2016	Italy	Journal article	Parallel	80	76.4	36.3	22.9	AD	Clinical
Yang^2^	2017	South Korea	Journal article	Parallel	20	71	70	23.1	AD	Clinical
Zhuang^3^	2013	China	Journal article	Parallel	33	83.1	24.2	10.2	MCI, dementia	Clinical
Leung^4^	2015	Hong Kong	Journal article	Parallel	209	70.1	21.5	NR	MCI	Community
Tarnanas^5^	2014	Greece	Book chapter	Parallel	114	70.3	39	26.4	MCI	Clinical
Herrera^6^	2012	France	Journal article	Parallel	22	76.6	50	27.3	MCI	Clinical
Flak^7^	2019	Norway	Journal article	Parallel	85	66	66.7	NR	MCI	Clinical
Park^8^	2017	South Korea	Journal article	Parallel	78	67.3	53.8	26.5	MCI	Community
Lee^9^	2018	South Korea	Journal article	Parallel	20	74.3	40	17.9	AD, MCI, dementia	Clinical
Hagovská^10^	2016	Slovakia	Journal article	Parallel	80	67	51.2	26.4	MCI	Clinical

*AD* Alzheimer’s disease, *MCI* mild cognitive disorder, *MMSE* Mini-Mental State Examination, *NR* not reported.

Ten distinct serious games were identified in the studies (Table 2). Cognitive training was the therapeutic modality used by serious games in all studies. In all trials, the games were built from the outset with a “serious” specific purpose (e.g., designed serious games). The platform of serious games was personal computers in most included studies (*n* = 9). Serious games were played under the supervision of caregivers or medical professionals in the majority of studies (*n* = 7). The duration of the serious games was 60 min or lower in most trials (*n* = 8). The games were played between two and five times a week, with two and three times a week being the most common in the studies (*n* = 8). The duration of the interventions ranged from 3 to 24 weeks. In 70% of the studies (7 out of 10), the intervention period was shorter than 13 weeks.

**Table 2:** Characteristics of interventions.

Study [Ref]	Intervention	Serious game name	Serious game type	Platform	Supervision	Duration (minute)	Frequency (time/week)	Period (week)
Cavallo^1^	Brainer	Cognitive training game	Designed	PC	Supervised	30	3	12
Yang^2^	Brain-Care	Cognitive training game	Designed	PC	Home-based	60	2	12
Zhuang^3^	NR	Cognitive training game	Designed	PC	Supervised	75	3	24
Leung^4^	BrainFitness	Cognitive training game	Designed	PC	Home-based	60	3	13
Tarnanas^5^	Virtual Reality Museum	Cognitive training game	Designed	VR headset	Supervised	90	2	21
Herrera^6^	NR	Cognitive training game	Designed	PC	Supervised	60	2	12
Flak^7^	Cogmed	Cognitive training game	Designed	PC	Home-based	30-40	5	5
Park^8^	CoTras	Cognitive training game	Designed	PC	Supervised	30	3	10
Lee^9^	Bettercog	Cognitive training game	Designed	PC	Supervised	30	4	3
Hagovská^10^	CogniPlus	Cognitive training game	Designed	PC	Both	30	2	10

*NR* not reported, *PC* personal computer, *VR* virtual reality.

The comparison groups in 50% of the trials (*n* = 5) either received no intervention or passive intervention (such as reading news articles, surfing the Internet, or watching a documentary program), while the comparison groups in six studies received active interventions (such as conventional exercises and other serious games). The number does not add up, as one study used both active and passive interventions as comparators (Table 3). The active comparators’ duration varied between 30 and 90 min. They were employed 2–7 times weekly. The timeframe for active comparators extended from 3 to 21 weeks. There were 11 different tools used to assess the outcome of interest (i.e., attention), with the Wechsler Memory Scale III-Digit Span Forwards (WMS-III-DSF) being the most commonly used among the included studies (*n* = 3). The outcomes were examined immediately after the intervention in all the studies included. Only three studies had a follow-up period to assess the outcomes. The number of participants who dropped out of the included studies was reported in nine studies and ranged from 0 to 17.

**Table 3:** Characteristics of comparators and outcomes.

Study [Ref]	Comparator	Duration (minute)	Frequency (time/week)	Period (week)	Measured outcomes	Outcome measures	Follow-up	Attrition
Cavallo^1^	Control	30	3	12	WMS-R-DSF	Postintervention, 24-week follow-up	4	NR
Yang^2^	Control	NA	NA	NA	WMS-III-DSF	Postintervention	0	10
Zhuang^3^	Control	NA	NA	NA	ACE-R-A	Postintervention	10	2
Leung^4^	Control	60	3	13	DVT, SRT	Postintervention	0	14
Tarnanas^5^	Control, Conventional cognitive activities	90	2	21	WMS-III-DSF	Postintervention	9	22
Herrera^6^	Conventional cognitive activities	60	2	12	WMS-R-DSF	Postintervention, 24-week follow-up	NR	15
Flak^7^	Serious games	30–40	5	5	WMS-III-DSF, WMS-III-SSF, CVLT-II-T1	Postintervention, 16-week follow-up	17	7
Park^8^	Serious games	30	3	10	WAIS-DSF	Postintervention	0	0
Lee^9^	Serious games	30	4	3	DSF, SNSB-II	Postintervention	1	4
Hagovská^10^	Conventional exercises	30	7	10	ACE-A	Postintervention	2	0

*ACE* Addenbrooke’s Cognitive Examination, *ACE-R* Addenbrooke’s Cognitive Examination-Revised, *CVLT-II-T1* California Verbal Learning Test II-Trail 1, *DVT* Digit Vigilance Test, *DSF* Digit span forward, *NA* not applicable, *NR* not reported, *SRT* Seashore Rhythm Test, *SNSB-II* The Seoul Neuropsychological Screening Battery 2nd edition, *WAIS-DSF* Wechsler Adult Intelligence Scale-Digit Span Forwards, *WMS-III-DSF* Wechsler Memory Scale III-Digit Span Forwards, *WMS-III-SSF* Wechsler Memory Scale III-Spatial Span forwards, *WMS-DSF* Wechsler Memory Scale-Digit Span Forwards, *WMS-R-DSF* Wechsler Memory Scale-Revised-Digit Span Forwards Test.

### Results of risk of bias appraisal

**Fig. 2:**
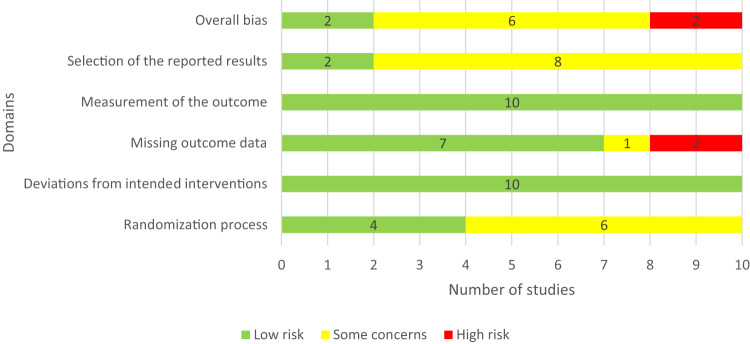
Results of the assessment of the risk of bias in the included studies. Risk-of-Bias 2 (RoB-2) tool was used to assess the risk of bias in five areas of RCTs: the randomization process, deviations from intended interventions, missing outcome data, evaluation of outcome, and selection of reported results. Low risk (green) refers to the number of studies that have a low risk of bias in the respective domain. Unclear (yellow) refers to the number of studies that have an unclear risk of bias in the respective domain due to a lack of information reported by the study. High risk (Red) refers to the number of studies that have a high risk of bias in the respective domain.

As depicted in Fig. 2, 40% (4 out of 10) of the studies were determined to have a minimal risk of bias in the “randomization process” domain. In terms of deviations from intended interventions and measurement of the outcome, all studies exhibited a low risk of bias. Concerning the “missing outcome data” category, a low risk of bias was identified in 70% (7 out of 10) of the studies. The risk of bias due to the selection of reported results was low in 20% (2 out of 10) of the studies. Based on these evaluations, only 20% (2 out of 10) of the studies were deemed to have a low risk of bias in the final domain (i.e., overall bias). The reviewers’ assessments for each “risk of bias” category for every included study can be found in Supplementary Fig. 1.

### Results of studies

#### Serious games versus no/passive interventions

**Fig. 3:**
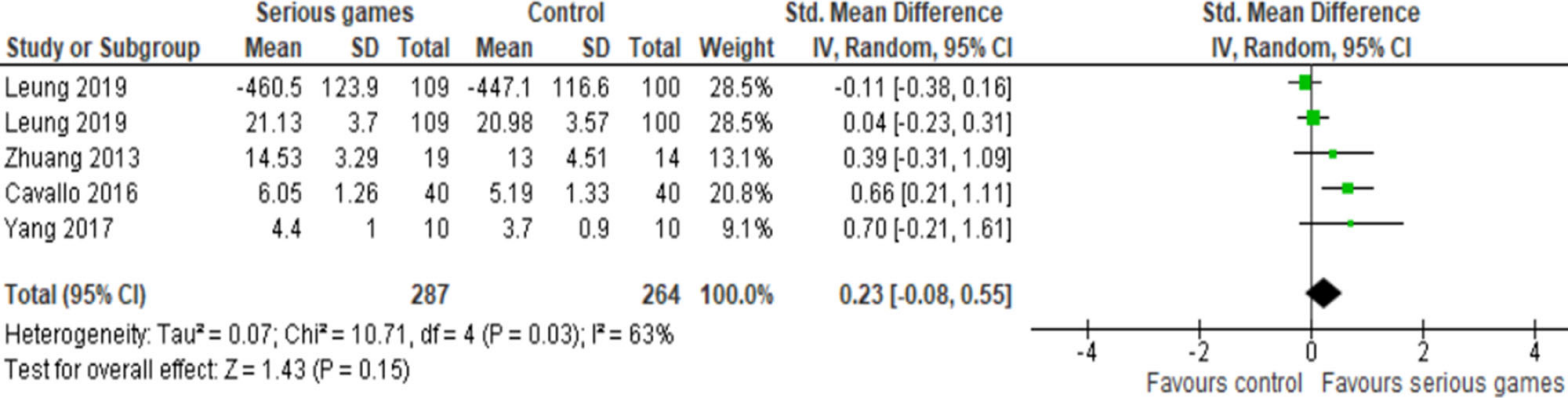
Forest plot of four studies (five comparisons) comparing the effect of serious games with control. The meta-analysis showed no statistically significant difference (*P* = 0.15) in attention between the “serious games” group and the “no/passive interventions” group (SMD 0.23; 95% confidence interval (CI) −0.08 to 0.55). The statistical heterogeneity of the evidence was substantial (*P* = 0.03; I^2^ = 63%).

In 50% of the studies (5 out of 10), the effect of serious games was compared to a control group, which consisted of either no intervention or passive interventions^29–33^. Passive interventions are those which are not known to influence the measured outcome, such as reading newspapers, browsing the internet, and watching documentary programs. One study among these was deemed to carry a high risk of bias and thus was not included in the meta-analysis^33^. One of the remaining four studies employed two distinct tools for attention assessment^32^, leading to our meta-analysis comprising five comparisons drawn from four studies (Fig. 3). The meta-analysis revealed no statistically significant difference (*P* = 0.15) in attention between the “serious games” group and the “no/passive interventions” group (SMD 0.23; 95% confidence interval (CI) −0.08 to 0.55). The statistical heterogeneity of the evidence was substantial (*P* = 0.03; I^2^ = 63%). The quality of evidence was deemed very low, having been downgraded by five levels due to high risk of bias, heterogeneity, and imprecision (Supplementary Table 1).

**Fig. 4:**
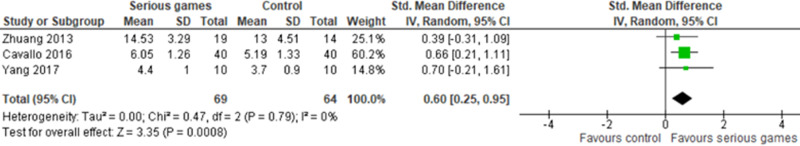
Forest plot of three studies comparing the effect of serious games with control. The sensitivity analysis showed a statistically significant difference (*P* < 0.001) in attention between groups, favoring serious games over no/passive interventions (SMD 0.60; 95% CI 0.25 to 0.95). This difference was also clinically important as the overall effect was outside MCID boundaries (−0.30 to 0.30), and its CI did not cross the “no effect” line (zero effect). The statistical heterogeneity of the evidence was not a concern (*P* = 0.79; I^2^ = 0%).

It seems that the results of one study^32^ were the source of the substantial heterogeneity in this meta-analysis. More specifically, the study had the lowest participants’ mean age and male percentage among all studies in this meta-analysis, and it is the only study that recruited participants from the community. As such, we conducted a sensitivity analysis to determine if the results of this particular study^32^ influenced the overall effect size and heterogeneity level (Fig. 4). The sensitivity analysis revealed a statistically significant difference (*P* < 0.001) in attention between the groups, with serious games performing better than no or passive interventions (SMD 0.60; 95% CI 0.25 to 0.95). This difference was also clinically meaningful, as the overall effect exceeded the MCID boundaries (−0.30 to 0.30), and its CI did not cross the “no effect” line (zero effect). The statistical heterogeneity of the evidence was not an issue (*P* = 0.79; I^2^ = 0%). However, the quality of the evidence was notably low, having been reduced by 3 levels due to a high risk of bias and imprecision (Supplementary Table 1).

#### Serious games versus conventional cognitive training

In two studies, the effect of serious games was compared with traditional cognitive training^33,34^. The first study^33^ demonstrated a statistically significant enhancement (*P* = 0.04) in attention among participants of the “serious games” group compared to those in the “conventional cognitive training” group. In line with this, the second study also found that serious games were superior to traditional cognitive training in boosting attention (*P* = 0.009). Due to the high risk of bias present in both studies, no meta-analysis was conducted.

#### Serious games versus other serious games

Three studies investigated the impact of different types of serious games on attention^35–37^. Due to the dissimilarity of serious games in the comparison groups across the studies, we did not conduct a meta-analysis to pool the results of these studies. One study^35^ compared an adaptive serious game, which adjusted the task difficulty based on the user’s proficiency at each level, with the same game but without such adjustments (referred to as a nonadaptive game). The study concluded that there is a comparable effect of these two games on attention as assessed by three different questionnaires^35^.

The second study contrasted two distinct forms of serious games: cognitive training games and exergames^36^. The study demonstrated that attention level in exergame group improved considerably in comparison with the cognitive training game group^36^. In the third study^37^, the researchers compared a serious game called COMCOG, which focuses solely on attention and memory, with another serious game called Bettercog, which focuses on a wide range of cognitive abilities, including attention, executive function, and visuospatial function. The study revealed that there is a comparable effect of these two games on attention as assessed by two different questionnaires^37^.

#### Serious games versus conventional exercises

Only one study^38^ compared the impact of serious games with that of traditional exercises. This study revealed a statistically significant difference in attention between the two groups, with serious games showing a better outcome than conventional exercises^38^.

## Discussion

The present study conducted a review of the evidence pertaining to the efficacy of serious games in enhancing attention in older adults with cognitive impairment. This review suggested that serious games can indeed aid in improving attention among this demographic. However, the conclusions were not definitive due to the low quality of evidence, small sample sizes in most included studies, absence of studies for some comparisons, and scarcity of studies included in the meta-analyses. Our results did not align with the findings of a previous review^25^, where the meta-analyses indicated that serious games are equally effective as passive and active interventions in promoting attention among the elderly^25^. The discrepancy in findings could be attributed to the fact that our study concentrated on older adults with cognitive impairment, while the study by Lampit et al.^25^ focused on older adults without cognitive impairment.

A previous review assessed the impact of serious games, specifically cognitive training games, compared to any intervention (passive and active) on attention in older adults^24^. The review, through a meta-analysis of four studies, showed no statistically significant difference between serious games and other interventions in terms of improving attention^24^. However, the review centered on older adults without cognitive impairment and did not contrast the effect of serious games with a specific comparator. Similarly, another review examined the impact of serious games, specifically virtual reality exergames, on attention among older adults compared to any intervention^27^. Through a meta-analysis of seven studies, the review found that serious games were not superior to other interventions in enhancing attention among older adults^27^. Still, this review also focused on older adults without cognitive impairment and did not compare the impact of serious games to a specific comparator.

Given the following reasons, it is crucial for readers of this review to approach our findings with caution: (1) The evidence quality was notably low, primarily because of the substantial risk of bias, imprecise estimation of pooled effect sizes, and significant heterogeneity; (2) The meta-analyses contained a limited number of studies, ranging from 2 to 5; (3) majority of studies in our meta-analyses had small sample sizes. Therefore, until more reliable evidence becomes available, it is advisable to view serious games as a supplementary intervention to existing interventions rather than a complete substitute.

Our results showed that serious games were mostly delivered through computers, whereas platforms such as mobile devices (e.g., smartphones and tablets) and virtual reality headsets were used in no or only one study, respectively. Intuitively, a serious game delivered through a virtual reality headset could capture more attention of the user than a serious game delivered through a computer since the latter does allow for more distraction through external factors. Further, mobile devices (e.g., tablets and smartphones) are usually cheaper, more pervasive, and more accessible than other platforms such as computers and gaming consoles; thereby, they are more appealing.

The global count of mobile devices and users stood around 15 billion and 7.1 billion, respectively, in 2021, with projections suggesting a significant increase by 2025^39^. This data highlights the need for game developers to focus on creating serious games that can be delivered via mobile devices and virtual reality headsets. Moreover, developers should aim to create serious games specifically designed to enhance attention, as none of the serious games included in the studies particularly targeted this cognitive function.

As more serious games are being produced for older adults, we need to develop a repository ranking the serious games and their impact on older adults. The databases should be categorized for their purpose and evaluated for their information, ease of use, and impact on a number of different outcomes that are relevant to the elderly needs. Such a database would be helpful for families and elderly facilities to select the most appropriate serious game intervention that would help support families caring for their elderly. Such a platform does not exist and would be very practical for older adults.

We recognized that there remains a limited amount of research that examines the impact of serious games in comparison with alternative interventions (e.g., traditional cognitive training and exercises). Additionally, very few or no studies have compared various genres of serious games (e.g., cognitive training games vs. exergames) and various platforms for serious games (e.g., computers vs. virtual reality headsets vs. mobile devices) in terms of their effects on attention. To fill these gaps, researchers should conduct more comprehensive studies, enabling us to substantiate the results with a larger body of evidence.

In most studies, there was no follow-up period, and the intervention period was short (≤3 months). This brings up a serious issue of the long-term impact of serious games on improving attention within the elderly population. It would be important to determine the progression and/or decline of attention over longer periods of time as the cognitive decline is time-dependent^40^. Therefore, further studies are needed to assess the long-term effect of serious games and to compare different intervention periods.

We noticed that no studies relevant to this review were published after 2019. By the time of the publication of this work, it would make the work with the highest evidence outdated—almost 5 years old. Thus, we urge researchers to update the evidence in this area. Most of the studies we considered were conducted in wealthy nations. Thus, our results might not apply to elderly individuals in less affluent countries. Therefore, it is crucial to urge for a more balanced research approach that involves low-income countries as much as high-income ones.

Most of the studies in this review did not supply the necessary data (mean and standard deviation) for changes in attention before and after interventions for each group. This information is vital for accurately determining effect sizes in our meta-analyses. Therefore, future trials should include such information in their reports. Out of all the studies examined, only two were deemed to have a low overall risk of bias. The rest of the studies exhibited concerns primarily related to randomizing participants into groups or selecting results reported in the study. To mitigate the risk of bias, it is crucial for future trials to be conducted in accordance with established guidelines like the RoB-2. This will help ensure that the potential for bias is minimized in subsequent research endeavors.

In a substantial number of studies, details regarding the psychological approach employed in serious games were noticeably absent. That is, it was not clear whether the serious games were developed based on psychological theories and clinical evidence. This data is crucial to facilitate a more comprehensive comparison between the impact of theory-based serious games and commercial serious games on attention. It is recommended that future research reports include such details for enhanced transparency and comparability.

This review is unable to offer insights on (1) the long-term effects of serious games, (2) the efficacy of non-digital platform serious games or those used for different purposes such as screening or diagnosis, (3) the impact of serious games on other cognitive abilities like memory and processing speed, and (4) how effective serious games are at enhancing attention in adolescents, young adults, middle-aged adults, and individuals without cognitive impairment. These aspects were not covered due to the defined boundaries of this review, which excluded these specific interventions, outcomes, and populations. This review did not leverage data concerning the change in attention before and after each intervention to calculate the effect size for each study. Rather, we relied on data after the intervention as only one study furnished the mean and standard deviation of the attention change for each group before and after the intervention. Moreover, most studies did not show a statistically significant difference in baseline attention levels between the groups. As a result, the effect sizes derived in our review may be subject to either overestimation or underestimation. Given that we excluded quasi-experiments, pilot RCTs, and studies published in non-English languages before 2010, it is possible that our review missed certain pertinent studies. There may be a concern about the internal validity of our findings due to the considerably low quality of evidence across all meta-analyses.

In conclusion, serious games have the potential to enhance attention in older adults with cognitive impairment. However, this conclusion may not be definitive. As a result, serious games should serve as a complementary approach to existing interventions rather than as a total replacement until the aforementioned gaps are addressed. More research is necessary to contrast serious games with traditional cognitive training, conventional exercises, various kinds of serious games, and different serious game platforms. Additionally, further investigation is required to examine the long-term impact of serious games and compare varying intervention durations.

## Methods

The authors have carried out and presented this study in line with the guidelines of the Preferred Reporting Items for Systematic Reviews and Meta-Analyses (PRISMA), as detailed in Supplementary Table 2^41^. The protocol for this review has been officially registered with the International Prospective Register of Systematic Reviews (PROSPERO) under the protocol ID: CRD42022348893.

### Search strategy

#### Search sources

The first author carried out search queries in several databases, including Google Scholar, IEEE Xplore, ACM Digital Library, EMBASE (through Ovid), MEDLINE (via Ovid), PsycINFO (through Ovid), CINAHL (via EBSCO), and Scopus on July 22, 2022. For Google Scholar, only the initial 10 pages, which is equivalent to the first 100 results, were taken into account for this review since it does not provide as powerful search tools as other databases, and it typically retrieves many publications automatically sorted according to their relevance. We limited our search in these databases to studies published from 2010 onward because, first, video games have significantly been developed in the last decade, and, second, previous reviews did not find any study published on similar topics before 2012^12,14,17^. Backward reference list checking was carried out by screening the reference lists of included publications and related reviews. Lastly, we evaluated the eligibility of studies that had referenced the publications included in our review (forward reference list checking).

#### Search terms

In order to develop the search query, we consulted with two experts in the field of digital mental health. The search terms were associated with the intended population (for instance, cognitive impairment), the intended intervention (like serious games), and the target study design (such as RCTs). We employed Medical Subject Headings (MeSH), term truncation, and wildcards as required. The specific search queries utilized for each of the eight databases are demonstrated in Supplementary Table 3.

### Study eligibility criteria

This review exclusively incorporated RCTs examining the efficacy of serious games in enhancing attention in older individuals suffering from cognitive impairment. We considered studies that employed serious games on various electronic platforms such as computers, gaming consoles, mobile phones, or tablets. The game should be the primary component of the intervention. Studies that combined serious games with additional interventions were also included, provided the control group received the same adjacent intervention. However, we did not consider serious games used for research, screening, diagnosis, or monitoring purposes or non-digital games such as board or card games.

To be eligible for this review, participants in studies should be older adults (60 years or older) with any form of cognitive impairment or disorder determined by well-established diagnostic standards (e.g., the Mini-Mental State Examination (MMSE). Studies on elderly people without cognitive impairment, healthcare professionals, or caregivers were not included in this review. There were no restrictions on the ethnicity or gender of the participants.

The main outcome this review was focused on was attention. We imposed no limitations on the outcome measures. Studies that solely evaluated usability, feasibility, satisfaction, cost-effectiveness, or other cognitive functions were not considered in this review. Attention measures that were assessed immediately after the intervention (i.e., postintervention data) were used in this paper. This review did not use follow-up data (i.e., attention measures collected after a period of the intervention), given that only two studies collected follow-up data.

This review did not consider pilot RCTs, observational studies, quasi-experiments, and reviews. All forms of RCTs, such as parallel, cluster, crossover, and factorial, were incorporated. Acceptable sources included dissertations, journal articles, and conference proceedings, while editorials, commentaries, proposals, posters, and conference abstracts were excluded. We only included studies published in English and dating from 2010 onward. There were no limitations concerning the research settings, the comparator, or the country of publication.

### Study selection

The methodology we adopted to ascertain the eligibility of the retrieved studies is outlined as follows: initially, all retrieved studies were uploaded into EndNote to identify and eliminate duplicate studies. Then, the titles and abstracts of all retrieved papers were independently assessed by two reviewers (the first and second authors). Lastly, the full text of the papers that were approved in the preceding step was independently evaluated by the two reviewers. Any discrepancies were resolved through discussions between the two reviewers. The interrater agreement, as measured by Cohen’s kappa (κ), for the second and third steps was 0.91 and 0.92, respectively.

### Data extraction

Two reviewers independently extracted data from the selected studies utilizing Microsoft Excel. Before proceeding with the extraction, a pilot test of the data extraction form was carried out using two of the included studies. Any disagreements were resolved by discussions between the two reviewers. Supplementary Table 4 presents the data extraction form employed to gather data from the chosen studies. If essential metrics, such as mean, standard deviation, and sample size, were not provided in the published paper, the first and corresponding authors were approached to provide this data.

### Risk of bias appraisal

The risk of bias in the included studies was independently assessed by two reviewers (the first and second authors) using the Risk-of-Bias 2 (RoB-2) tool^42^. This tool examines the risk of bias across five key domains of RCTs: the randomization process, deviations from intended interventions, missing outcome data, evaluation of the outcome, and selection of reported results^39^. Any differences in the risk of bias assessment were addressed through discussions between the reviewers, and the resulting interrater agreement was 0.89.

### Data synthesis

Both narrative and statistical approaches were used to summarize the data from the included trials. With text and tables, narrative synthesis was used to summarize the meta-data of the study, the characteristics of the interventions, participants, comparisons, and outcomes. The experimental findings were compiled and categorized according to the comparator, which consisted of no interventions or passive interventions, traditional cognitive training, standard exercises, and other serious games. In alignment with other reviews, meta-analyses were conducted using Review Manager (RevMan 5.4) when two or more studies from the same comparator provided sufficient data (that is, the number of participants in each intervention group, mean, standard deviation)^12,14,17^. Studies evaluated to carry a high risk of bias were excluded from the meta-analyses. The primary outcome of interest, attention, was treated as a continuous variable across all studies, which utilized a variety of tools for outcome assessment. Consequently, the standardized mean difference (SMD; Cohen’s d) was applied to estimate the overall effect of each trial. SMD is a unitless measure of effect size that allows for the comparison of effect sizes across different studies and different measures. We selected the “random effects” model in the analysis due to the significant clinical heterogeneity of meta-analyzed studies in terms of population characteristics (e.g., sample size, mean age, and health condition), serious game attributes (e.g., types, duration, frequency, and period), and outcome measures (i.e., tools and follow-up periods).

In cases where a statistically significant variance between groups emerged in a meta-analysis, we verified whether this difference is clinically significant. The concept of “minimal clinically important difference” (MCID) alludes to the minimum alteration in an evaluated outcome that a patient would perceive as valuable and significant enough to justify a shift in treatment^43^. The boundaries for the MCID were determined as ±0.5 times the SMD of the studies included in the meta-analysis.

In order to evaluate the extent of heterogeneity and its statistical significance within the meta-analyzed studies, we computed the I^2^ statistic and a chi-square *P*-value. A chi-square *P*-value of ≤0.05 suggests the presence of heterogeneity in the meta-analyzed studies^44^. The extent of heterogeneity was deemed insignificant if I^2^ was between 0 and 40%, moderate between 30 and 60%, substantial between 50 and 90%, and considerable between 75 and 100%^44^.

The authors employed the Grading of Recommendations Assessment, Development, and Evaluation (GRADE) approach to evaluate the overall quality of evidence derived from the meta-analyses^45^ (Supplementary Table 1). The GRADE approach assesses the quality of evidence based on five criteria: publication bias, indirectness, imprecision, inconsistency (i.e., heterogeneity), and risk of bias^45^. The quality of evidence was independently evaluated by two reviewers (the first and second authors), with any disagreements between them resolved through discussions. The interrater agreement between the reviewers was determined to be 0.90.

### Reporting summary

Further information on research design is available in the Nature Research Reporting Summary linked to this article.

### Supplementary information

SUPPLEMENTAL MATERIAL

Reporting Summary

## Data Availability

The corresponding author can be contacted to request access to the data supporting the findings of this study.

## Code availability

This study did not involve the utilization of any custom code or mathematical algorithms.
