# Supplementary material for: The performance of serious games for enhancing attention in cognitively impaired older adults
Source: NPJ Digit Med. 2023 Jul 8;6:122. doi: 10.1038/s41746-023-00863-2 (PMC10329640; doi:10.1038/s41746-023-00863-2)
Supplement: Supplementary file 1 — SUPPLEMENTAL MATERIAL [file 41746_2023_863_MOESM1_ESM.pdf]

Supplementary Figure 1: Reviewers' judgments about each "risk of bias" domain for each included study

Study ID	D1	D2	D3	D4	D5	Overall
Cavallo 2016	!	+	+	+	!	!
Yang 2017	!	+	+	+	!	!
Zhuang 2013	!	+	!	+	!	!
Leung 2015	!	+	+	+	!	!
Tarnanas 2014	!	+	-	+	!	-
Herrera 2012	!	+	-	+	!	-
Flak 2019	+	+	+	+	+	+
Park 2018	+	+	+	+	+	+
Lee 2018	+	+	+	+	!	!
Hagovska 2016	+	+	+	+	!	!

+	Low risk
!	Some concerns
-	High risk

D1	Randomisation process
D2	Deviations from the intended interventions
D3	Missing outcome data
D4	Measurement of the outcome
D5	Selection of the reported result

Supplementary Table 1: GRADE Profile for comparison of serious games to control for attention											
Certainty assessment							Summary of findings				
Participants (studies) Follow-up	Risk of bias	Inconsistency	Indirectness	Imprecision	Publication bias	Overall certainty of evidence	Study event rates (%)		Relative effect (95% CI)	Anticipated absolute effects	
							Control	Serious games		Risk with placebo	Risk difference with Serious games
**Serious games vs. Control**											
551 (4 RCTs) (5 comparisons)	very serious[a]	very serious[b]	not serious	serious[c,d]	none	⊕◯◯◯ Very low	264	287	-	-	SMD 0.23 higher (0.08 lower to 0.55 higher)
**Serious games vs. Control (sensitivity analysis)**											
133 (3 RCTs)	very serious[e]	not serious	not serious	serious[c,f]	none	⊕◯◯◯ Very low	64	69	-	-	SMD 0.60 higher (0.25 higher to 0.95 higher)

CI: confidence interval; SMD: standardised mean difference

Explanations

a. Evidence was downgraded by 2 levels because none of the meta-analyzed studies in this comparison was judged to have a low risk of bias, this is due to issues mainly in the randomization process and selection of the reported results.

b. Evidence was downgraded by 2 levels as P=0.03 and I square=63%, indicating high and statistically significant heterogeneity.

c. Evidence was downgraded by 1 level because 95% CI crosses one of the two minimally important differences (MID) boundaries for this outcome.

d. MID for this outcome, calculated as ± 0.5 times the standardized mean difference (SMD), is ± 0.115.

e. Evidence was downgraded by 2 levels because none of the meta-analyzed studies in this comparison was judged to have a low risk of bias, this is due to issues mainly in the randomization process and selection of the reported results.

f. MID for this outcome, calculated as ± 0.5 times the standardized mean difference (SMD), is ± 0.30.

Supplementary Table 2: PRISMA checklist

Section/topic	#	Checklist item	Reported on page #
TITLE			
Title	1	Identify the report as a systematic review, meta-analysis, or both.	1
ABSTRACT			
Structured summary	2	Provide a structured summary including, as applicable: background; objectives; data sources; study eligibility criteria, participants, and interventions; study appraisal and synthesis methods; results; limitations; conclusions and implications of key findings; systematic review registration number.	2
INTRODUCTION			
Rationale	3	Describe the rationale for the review in the context of what is already known.	3 & 4
Objectives	4	Provide an explicit statement of questions being addressed with reference to participants, interventions, comparisons, outcomes, and study design (PICOS).	4
METHODS			
Protocol and registration	5	Indicate if a review protocol exists, if and where it can be accessed (e.g., Web address), and, if available, provide registration information including registration number.	5
Eligibility criteria	6	Specify study characteristics (e.g., PICOS, length of follow-up) and report characteristics (e.g., years considered, language, publication status) used as criteria for eligibility, giving rationale.	5 & 6
Information sources	7	Describe all information sources (e.g., databases with dates of coverage, contact with study authors to identify additional studies) in the search and date last searched.	5
Search	8	Present full electronic search strategy for at least one database, including any limits used, such that it could be repeated.	5 & Appendix 2
Study selection	9	State the process for selecting studies (i.e., screening, eligibility, included in systematic review, and, if applicable, included in the meta-analysis).	6
Data collection process	10	Describe method of data extraction from reports (e.g., piloted forms, independently, in duplicate) and any processes for obtaining and confirming data from investigators.	6
Data items	11	List and define all variables for which data were sought (e.g., PICOS, funding sources) and any assumptions and simplifications made.	6 and Appendix 3
Risk of bias in individual studies	12	Describe methods used for assessing risk of bias of individual studies (including specification of whether this was done at the study or outcome level), and how this information is to be used in any data synthesis.	7
Summary measures	13	State the principal summary measures (e.g., risk ratio, difference in means).	7 & 8
Synthesis of results	14	Describe the methods of handling data and combining results of studies, if done, including measures of consistency (e.g., I^2) for each meta-analysis.	7 & 8

Supplementary Table 2: PRISMA checklist

Section/topic	#	Checklist item	Reported on page #
Risk of bias across studies	15	Specify any assessment of risk of bias that may affect the cumulative evidence (e.g., publication bias, selective reporting within studies).	8
Additional analyses	16	Describe methods of additional analyses (e.g., sensitivity or subgroup analyses, meta-regression), if done, indicating which were pre-specified.	-
RESULTS			
Study selection	17	Give numbers of studies screened, assessed for eligibility, and included in the review, with reasons for exclusions at each stage, ideally with a flow diagram.	8 & 9
Study characteristics	18	For each study, present characteristics for which data were extracted (e.g., study size, PICOS, follow-up period) and provide the citations.	10-15
Risk of bias within studies	19	Present data on risk of bias of each study and, if available, any outcome level assessment (see item 12).	15 & 16
Results of individual studies	20	For all outcomes considered (benefits or harms), present, for each study: (a) simple summary data for each intervention group (b) effect estimates and confidence intervals, ideally with a forest plot.	16-21
Synthesis of results	21	Present results of each meta-analysis done, including confidence intervals and measures of consistency.	16-21
Risk of bias across studies	22	Present results of any assessment of risk of bias across studies (see Item 15).	16-21
Additional analysis	23	Give results of additional analyses, if done (e.g., sensitivity or subgroup analyses, meta-regression [see Item 16]).	-
DISCUSSION			
Summary of evidence	24	Summarize the main findings including the strength of evidence for each main outcome; consider their relevance to key groups (e.g., healthcare providers, users, and policy makers).	22 & 23
Limitations	25	Discuss limitations at study and outcome level (e.g., risk of bias), and at review-level (e.g., incomplete retrieval of identified research, reporting bias).	24 & 25
Conclusions	26	Provide a general interpretation of the results in the context of other evidence, and implications for future research.	28
FUNDING			
Funding	27	Describe sources of funding for the systematic review and other support (e.g., supply of data); role of funders for the systematic review.	29

From: Moher D, Liberati A, Tetzlaff J, Altman DG, The PRISMA Group (2009). Preferred Reporting Items for Systematic Reviews and Meta-Analyses: The PRISMA Statement. PLoS Med 6(7): e1000097. doi:10.1371/journal.pmed1000097

For more information, visit: **www.prisma-statement.org**.

Supplementary Table 3: Search strategy

Database(s): **Ovid MEDLINE(R) ALL** 1946 to July 22, 2022
Search Strategy:

#	Searches	Results
1	exp Cognitive Dysfunction/	31732
2	"cognitive impair*".tw.	80393
3	"cognitive disorder*".tw.	5114
4	"Cognitive Dysfunction".tw.	16725
5	exp Dementia/	194718
6	dementia*.tw.	128477
7	exp Alzheimer Disease/	111786
8	Alzheimer*.tw.	170219
9	exp Randomized Controlled Trial/	576844
10	"randomized controlled trial*".tw.	174605
11	"randomised controlled trial*".tw.	52984
12	"randomized control trial*".tw.	9393
13	"randomised control trial*".tw.	2378
14	"clinical trial*".tw.	445369
15	experiment*.tw.	2307598
16	exp Video Games/	6871
17	"serious gam*".tw.	1024
18	"game-based".tw.	956
19	"videogame*".tw.	858
20	"video game*".tw.	4033
21	"virtual reality game*".tw.	146
22	"virtual reality-based game*".tw.	3
23	"Augmented Reality-based game*".tw.	0
24	"Augmented Reality game*".tw.	39
25	"gamification".tw.	901
26	exergame*.tw.	682
27	"Applied game*".tw.	25
28	1 or 2 or 3 or 4 or 5 or 6 or 7 or 8	356815
29	9 or 10 or 11 or 12 or 13 or 14 or 15	3306330
30	16 or 17 or 18 or 19 or 20 or 21 or 22 or 23 or 24 or 25 or 26 or 27	11348
31	28 and 29 and 30	117
32	limit 31 to (english language and yr="2010 -Current")	115

Database(s): **Embase** 1996 to 2022 Week 29
Search Strategy:

#	Searches	Results
1	exp Cognitive Dysfunction/	518736
2	"cognitive impair*".tw.	119588
3	"cognitive disorder*".tw.	7919
4	"Cognitive Dysfunction".tw.	24015
5	exp Dementia/	371510
6	dementia*.tw.	167586
7	exp Alzheimer Disease/	209750
8	Alzheimer*.tw.	212437

9	exp Randomized Controlled Trial/	673843
10	"randomized controlled trial*".tw.	220745
11	"randomised controlled trial*".tw.	69587
12	"randomized control trial*".tw.	14294
13	"randomised control trial*".tw.	3824
14	"clinical trial*".tw.	595130
15	experiment*.tw.	2056485
16	exp Video Games/	5304
17	"serious gam*".tw.	1080
18	"game-based".tw.	996
19	"videogame*".tw.	1026
20	"video game*".tw.	4800
21	"virtual reality game*".tw.	189
22	"virtual reality-based game*".tw.	4
23	"Augmented Reality-based game*".tw.	0
24	"Augmented Reality game*".tw.	33
25	"gamification".tw.	953
26	exergame*.tw.	673
27	"Applied game*".tw.	23
28	1 or 2 or 3 or 4 or 5 or 6 or 7 or 8	584271
29	9 or 10 or 11 or 12 or 13 or 14 or 15	3269176
30	16 or 17 or 18 or 19 or 20 or 21 or 22 or 23 or 24 or 25 or 26 or 27	11099
31	28 and 29 and 30	190
32	limit 31 to (english language and yr="2010 -Current")	187
33	limit 32 to exclude medline journals	38

Database(s): **APA PsycInfo** 2002 to July Week 2 2022
Search Strategy:

#	Searches	Results
1	exp Cognitive Dysfunction/	40610
2	"cognitive impair*".tw.	40370
3	"cognitive disorder*".tw.	2720
4	"Cognitive Dysfunction".tw.	6513
5	exp Dementia/	68783
6	dementia*.tw.	57350
7	exp Alzheimer Disease/	41793
8	Alzheimer*.tw.	56129
9	exp Randomized Controlled Trial/	1234
10	"randomized controlled trial*".tw.	31952
11	"randomised controlled trial*".tw.	6419
12	"randomized control trial*".tw.	2388
13	"randomised control trial*".tw.	441
14	"clinical trial*".tw.	36024
15	experiment*.tw.	280946
16	exp Video Games/	8246
17	"serious gam*".tw.	1084
18	"game-based".tw.	1795
19	"videogame*".tw.	1073
20	"video game*".tw.	5860

21	"virtual reality game*".tw.	78
22	"virtual reality-based game*".tw.	1
23	"Augmented Reality-based game*".tw.	0
24	"Augmented Reality game*".tw.	52
25	"gamification".tw.	1018
26	exergame*.tw.	359
27	"Applied game*".tw.	21
28	1 or 2 or 3 or 4 or 5 or 6 or 7 or 8	127298
29	9 or 10 or 11 or 12 or 13 or 14 or 15	347596
30	16 or 17 or 18 or 19 or 20 or 21 or 22 or 23 or 24 or 25 or 26 or 27	12881
31	28 and 29 and 30	46
32	limit 31 to (english language and yr="2010 -Current")	43

Database(s): **CINHAL (EBSCO)**

#	Query	Results
S1	SU cognitive impairment OR TI "cognitive impair*" OR AB "cognitive impair*"	25895
S2	SU cognitive dysfunction [mesh] OR TI "Cognitive Dysfunction" OR AB "Cognitive Dysfunction"	3670
S3	SU cognitive disorder* OR TI "cognitive disorder*" OR AB "cognitive disorder*	486
S4	SU Dementia OR TI Dementia OR AB Dementia	68506
S5	SU alzheimer's disease OR TI alzheimer's disease OR AB alzheimer's disease	43616
S6	SU Randomized Controlled Trials OR TI "Randomized Controlled Trial*" OR AB "Randomized Controlled Trial"	148187
S7	TI "Randomised Controlled Trial*" OR AB "Randomised Controlled Trial*"	25862
S8	TI "Randomized Control Trial*" OR AB "Randomized Control Trial*"	4352
S9	TI "Randomised Control Trial*" OR AB "Randomised Control Trial*"	1159
S10	TI "clinical trial*" OR AB "clinical trial*"	119312
S11	TI experiment* OR AB experiment*	146423
S12	SU Video Games OR TI "Video Game*" OR "Video Game*"	5884
S13	SU serious games OR TI "serious gam*" OR AB "serious gam*"	432
S14	TI "game-based" OR AB "game-based"	459
S15	TI "videogame*" OR AB "videogame*"	323
S16	TI "virtual reality game*" OR AB "virtual reality game*"	68
S17	TI "Augmented Reality-based game*" OR AB "Augmented Reality-based game*"	0
S18	TI "Augmented Reality game*" OR AB "Augmented Reality game*"	22
S19	TI "gamification" OR AB "gamification"	408
S20	TI exergam* OR AB exergam*	402
S21	TI "Applied game*" OR AB "Applied game*"	10
S22	S1 OR S2 OR S3 OR S4 OR S5	115589
S23	(S6 OR S7 OR S8 OR S9 OR S10 OR S11)	393430
S24	S12 OR S13 OR S14 OR S15 OR S16 OR S17 OR S18 OR S19 OR S20 OR S21	7102
S25	(S12 OR S13 OR S14 OR S15 OR S16 OR S17 OR S18 OR S19 OR S20 OR S21) AND (S22 AND S23 AND S24)	39
S26	Limiters - English Language (S25)	31

Database	Query	Results
Scopus	(TITLE-ABS-KEY ("serious gam*" OR "game-based" OR "videogame*" OR "video game*" OR "virtual reality game*" OR "virtual reality-based game*" OR "Augmented Reality-based game*" OR "Augmented Reality game*" OR "gamification" OR gamified OR exergam* OR "Applied game*") AND TITLE-ABS-KEY ("cognitive impair*" OR "cognitive disorder*" OR "Cognitive Dysfunction" OR dementia* OR alzheimer*) AND TITLE-ABS-KEY ("randomized controlled trial*" OR "randomised controlled trial*" OR "randomized control trial*" OR "randomised control trial*" OR "clinical trial*" OR experiment*)) AND (LIMIT-TO (PUBYEAR , 2021) OR LIMIT-TO (PUBYEAR , 2020) OR LIMIT-TO (PUBYEAR , 2019) OR LIMIT-TO (PUBYEAR , 2018) OR LIMIT-TO (PUBYEAR , 2017) OR LIMIT-TO (PUBYEAR , 2016) OR LIMIT-TO (PUBYEAR , 2015) OR LIMIT-TO (PUBYEAR , 2014) OR LIMIT-TO (PUBYEAR , 2013) OR LIMIT-TO (PUBYEAR , 2012) OR LIMIT-TO (PUBYEAR , 2011) OR LIMIT-TO (PUBYEAR , 2010)) AND (LIMIT-TO (LANGUAGE , "English")) AND (LIMIT-TO (DOCTYPE , "ar") OR LIMIT-TO (DOCTYPE , "cp"))	172
IEEE Xplore	("Abstract":"serious game" OR "Abstract":"serious games" OR "Abstract":"game-based" OR "Abstract":"videogames" OR "Abstract":"video games" OR "Abstract":"videogame" OR "Abstract":"video game" OR "Abstract":"virtual reality game" OR "Abstract":"virtual reality games" OR "Abstract":"Augmented Reality game" OR "Abstract":"Augmented Reality games" OR "Abstract":"gamification" OR "Abstract":gamified OR "Abstract":exergam* OR "Abstract":"Applied game") AND ("Abstract":"cognitive impair*" OR "Abstract": "cognitive disorder*" OR "Abstract": "Cognitive Dysfunction" OR "Abstract": dementia OR "Abstract": alzheimer*)	52
ACM Digital Library	*[[Abstract: "cognitive impair*"] OR [Abstract: "cognitive disorder*"] OR [Abstract: "cognitive dysfunction"] OR [Abstract: dementia*] OR [Abstract: alzheimer*]] AND [[Abstract: "serious gam*"] OR [Abstract: "game-based"] OR [Abstract: "videogame*"] OR [Abstract: "video game*"] OR [Abstract: "virtual reality game*"] OR [Abstract: "virtual reality-based game*"] OR [Abstract: "augmented reality-based game*"] OR [Abstract: "augmented reality game*"] OR [Abstract: "gamification"] OR [Abstract: gamified] OR [Abstract: exergam*] OR [Abstract: "applied game*"]] AND [[All: "randomized controlled trial*"] OR [All: "randomised controlled trial*"] OR [All: "randomized control trial*"] OR [All: "randomised control trial*"] OR [All: "clinical trial*"] OR [All: experiment*]] AND [Publication Date: (01/01/2010 TO 12/31/2021)]*	8

Google Scholar	("cognitive impair*" OR "cognitive disorder*" OR "Cognitive Dysfunction" OR dementia* OR alzheimer*) AND ("serious gam*" OR "game-based" OR exergam*) AND ("controlled trial*" OR "control trial*")	100

Supplementary Table 4 Data extraction form

Concept	Definition
Study Characteristics	
Author	The first author of the study.
Year of publication	The year in which the study was published.
Country of publication	The country where the study was published.
Type of publication	The venue where the study was published: peer-reviewed journal articles, book chapters, dissertations, or conference proceedings
Type of RCT	The type of the RCT used in the study (e.g., parallel, crossover, cluster, or factorial).
Population characteristics	
Number of participants	Number of people who participated in the study.
Number of participants in intervention group 1	Number of participants in intervention group 1.
Number of participants in intervention group 2 (if any)	Number of participants in intervention group 2 if there is more than one intervention.
Number of participants in the control group	Number of participants in the control group.
Mean age	The average age of participants.
Sex (male)	Percentage of males in the sample.
Health condition of participants	What is the health condition of the participants?
Recruitment setting	Place where participants were recruited (e.g., educational, clinical, community).
Intervention characteristics	
Name of the serious game	The name given for the serious game (e.g., SPARX, Tetris, etc..).
Therapeutic modality	What is the therapy that the serious game provides?
Serious game type	What is the type of serious games?<br>1. Designed serious games: games that are designed with a "serious" purpose from the beginning.<br>2. Purpose-shifted serious games: games that were not designed as serious games but are being used for a serious purpose.<br>3. Modified serious games: games that are similar to purpose-shifted ones, but while purpose-shifted games are left intact, modified ones can differ from the original in terms of gameplay and characters.
Platform	The platform in which the serious game is implemented (e.g., mobile, tablet, PC, Console, wearable devices, etc..).
Duration	How long does a session of playing the serious game take, e.g., 30 mins, 120 mins, etc?
Frequency	How many times the serious game was used per day or week, e.g., 3 times a week, 10 times a week.
Period	How long did the participants use the serious games (e.g., for 2 months, 6 months)?
Comparator Characteristics	

Supplementary Table 4 Data extraction form

Comparator	What is the comparator (e.g., usual care, waiting list, not intervention, giving information)?
Duration	How long does the comparator take, e.g., 30 mins, 120 mins, etc?
Frequency	How many times the comparator was used per day or week, e.g., 3 times a week, 10 times a week.
Period	How long did the participants use the comparator (e.g., for 2 months, 6 months).
Outcome characteristics	
Measured outcome	What was the outcome that the study measured?<br>Effectiveness: Attention
Outcome measure	What is the tool used for measuring the outcome?
Follow-up period	When was the outcome measured?
Attrition	Number of the loss/dropout of participants during an experiment.
Findings	
Results- Intervention- before: Mean (SD)	Results related to the outcome before delivering the intervention in the intervention group.
Results- control- before: Mean (SD)	Results related to the outcome before delivering the comparator in the control group.
Results- Intervention- after: Mean (SD)	Results related to the outcome after delivering the intervention in the intervention group.
Results- control- after: Mean (SD))	Results related to the outcome after delivering the comparator in the control group.
